# Going That Extra Mile: Individuals Travel Further to Maintain Face-to-Face Contact with Highly Related Kin than with Less Related Kin

**DOI:** 10.1371/journal.pone.0053929

**Published:** 2013-01-25

**Authors:** Thomas V. Pollet, Sam G. B. Roberts, Robin I. M. Dunbar

**Affiliations:** 1 Department of Social and Organizational Psychology, VU University Amsterdam, Amsterdam, The Netherlands; 2 Department of Psychology, University of Chester, Chester, United Kingdom; 3 Department of Experimental Psychology, University of Oxford, Oxford, United Kingdom; Université de Strasbourg, France

## Abstract

The theory of inclusive fitness has transformed our understanding of cooperation and altruism. However, the proximate psychological underpinnings of altruism are less well understood, and it has been argued that emotional closeness mediates the relationship between genetic relatedness and altruism. In this study, we use a real-life costly behaviour (travel time) to dissociate the effects of genetic relatedness from emotional closeness. Participants travelled further to see more closely related kin, as compared to more distantly related kin. For distantly related kin, the level of emotional closeness mediated this relationship - when emotional closeness was controlled for, there was no effect of genetic relatedness on travel time. However, participants were willing to travel further to visit parents, children and siblings as compared to more distantly related kin, even when emotional closeness was controlled for. This suggests that the mediating effect of emotional closeness on altruism varies with levels of genetic relatedness.

## Introduction

Inclusive fitness theory [1] has proved fundamental in explaining patterns of cooperation and altruism across a wide range of species [2], [3], including humans [4], [5] Hamilton's rule of kin selection states that a behaviour or trait will be favoured by selection when r*B>C, where C is the fitness cost to the actor, B is the fitness benefit to the recipient and *r* is the coefficient of genetic relatedness – the probability that two individuals share the same genes by descent [1]. Since the benefit of an action to the recipient is weighted by the coefficient of genetic relatedness, all other things being equal, more closely related individuals (with a higher *r*) are predicted to be favoured over less closely related individuals (with a lower *r*). In line with this theory's predictions, people offer greater levels of support to more closely related kin members, both in hypothetical (e.g., [6]–[8]) and real-life situations (e.g., [9], [10]).

However, whilst people broadly appear to act in line with inclusive fitness theory in distributing support amongst kin, the proximate psychological mechanisms underlying this behaviour are much less clear. In particular, the extent to which these fine-grained distinctions between kin of different relatedness are driven directly by knowledge of the genetic relatedness, and the degree to which they are mediated by other relationship variables, is a matter of intense debate [11]–[13]. Korchmaros and Kenny [13], [14] argue that emotional closeness is an important proximal cause for altruism towards kin. Emotional closeness is a widely used concept in social psychology [12]–[17], and previous definitions of emotional closeness have included concepts such as a sense of shared experience, concern for and trust of another individual, enjoyment of the relationship [18] and a feeling of support, willingness and confidence to disclose very personal feelings and the explicit willingness to place value on the relationship [17]. People tend to spend time, have frequent contact with and therefore form emotionally close relationships with more closely related kin, and Korchmaros and Kenny presented evidence that the level of emotional closeness mediates the relationship between kinship categories (which were then converted into genetic relatedness) and altruism [13], [14]. In contrast, other findings have led to suggestions that there may be a ‘kinship premium’ [12], in that kinship (as a purely linguistic label that correlates with true biological kinship) makes a significant unique contribution to altruism, even after controlling for the effects of emotional closeness (see also [19]).

Further, previous studies investigating the psychological mechanisms underpinning kinship have tended to treat ‘kinship’ as a unified category [11], [13], [20]–[22], in that the same mechanisms (e.g. the kinship premium, mediation via emotional closeness) are proposed to operate equally across all kin categories. However, given the fundamental importance of the coefficient of relatedness in shaping behaviour towards kin, it is possible that the mechanisms operate in distinct ways for different categories of kin. For example, a long period of co-residence, or maternal perinatal association, may trigger distinct psychological mechanisms towards parents and siblings (*r* = 0.5) that are not triggered for less closely related kin (e.g., [23]). Thus, the mediation with emotional closeness [13], [14] or the kinship premium [12] may not operate in a uniform way across all kin, but operate differently in kin with different degrees of relatedness.

The purpose of this study is to test whether emotional closeness mediates the relationship between genetic relatedness and investment in a kin relationship, using a real-life costly behaviour (distance travelled to visit a relative). We also examine whether emotional closeness mediates the relationship between genetic relatedness and investment in a kin relationship in the same way for kin with different levels of relatedness. Previous research that has controlled for the effect of emotional closeness on investment in kin has typically been based on hypothetical scenarios (for example [6], [8], [24]), raising doubts as to how well such responses mirror actual behaviour [25], [26]. Where research has been based on real-life behaviour [27], [28], it is unclear whether the results were driven directly by genetic relatedness (i.e. individuals lend support to others solely based on their kinship classification – parent, sibling, cousin etc.) or indirectly by emotional closeness, since these have invariably been confounded [29].

In this study, we combine these two approaches and examine how physical separation influences willingness to travel to see genetic relatives as a function of relatedness, and whether this is mediated by emotional closeness. Travel time has a genuine cost: travelling further costs more both in time (which is an inelastic resource, [30]) and also money. Although it is likely that both parties benefit from meeting, the cost of travel is asymmetric between the two parties. Hence, although we could frame our study either as a case of cooperative investment (both parties invest to facilitate cooperation) [31] or, given the positive effects of social relationships on health and well-being [32], a case of altruism (one party pays a cost to benefit the other [33]), depending on how the benefits are costed, we prefer to focus explicitly on the asymmetry in costs paid. Thus, in this study, we simply examine whether individuals are prepared to pay higher costs to visit some relatives, as opposed to others, in order to keep the relationship active. Keeping kin relationships active has been found to have positive effects on the financial and emotional support for, as well as the health of, one's offspring [34]. We hypothesize that people will travel further to see more closely related kin, than more distantly related kin. We also examine whether this relationship is mediated by emotional closeness. If investment in a relationship is mediated by emotional closeness, then after controlling for emotional closeness there should be no significant effect of genetic relatedness on willingness to travel. In contrast, if there is a ‘kinship premium’ [12] there should still be a significant effect of genetic relatedness on willingness to travel, even after controlling for emotional closeness.

## Materials and Methods

### Participants

355 participants, 72% German and 28% Dutch, were recruited via the University of Groningen. The sample consisted of 67% women, with a mean age of 29 years (*SD* = 13.63 years). Most of the respondents did not (yet) have a university degree (85% without university degree).

### Procedure

In order to obtain a larger non-student sample, participants were recruited via the personal networks of students. This method has been used successfully in previous studies (e.g. [15], [35]). Students completed a questionnaire and were instructed to hand out surveys to friends, colleagues and family. Surveys were returned in sealed envelopes. In order to avoid potential overlap, students were instructed not to hand out surveys to other students from the same degree as themselves. Students received course credits in return for completing this task (return rate >85%). The study was approved by the psychology ethics committee where the study was carried out.

### Questionnaire

Participants first provided some basic sociodemographic data, including age, gender, nationality and educational attainment. They then listed the initials of all their living relatives and specified their kin category (parent, child, full sibling, etc.), and whether they were biological kin, step, affinal or adopted. Given that less than 2.5% of relatives listed were step, affinal or adopted, we excluded these categories. Our sample thus only contained biological kinship ties (4,867 kinship ties). On average, individuals listed 14 biological kin (*SD* = 8.37).

For each reported biological relationship, we coded the coefficient of relatedness [36]. There were four categories: *r* = 0.5 (siblings, parents, children), *r* = 0.25 (half-siblings, nieces/nephews, grandparents, uncles and aunts), *r* = 0.125 (first cousins, great-grandparents, great-uncles, great-aunts), *r* = 0.03125 (second cousins).

Participants classified their last face-to-face contact with each relative in terms of six categories (within last 2 days (1); 3–7 days ago (2); 8–14 days ago (3); 15–30 days ago (4); over a month ago (5); never (6)). Previous work on social networks has consistently identified at least two distinct types of social ties (sometimes called ‘core’ and ‘significant’ ties), with differing degrees of emotional strength and which offer differing degrees of emotional and material support [37]–[39]. These closest core ties have also been referred to as the ‘support group’, which consists of around five individuals, from whom one would solicit help in times of personal crisis [40]–[42]. The less emotional intense significant ties have been referred to as the ‘sympathy group’, which consists of around fifteen individuals, inclusive of the support group, and can be defined as those individuals whose sudden death would be greatly upsetting [42], [43]. In turn, the support and sympathy group overlap roughly with weekly and monthly face-to-face contact circles [35], [40].

Since these two groups of social ties have different properties, it is possible that emotional closeness may mediate the relationship between kinship and willingness to travel differently in the support and sympathy groups. For example, it may be that willingness to travel is more reliant on kinship for the weaker ties in the sympathy group, but more dependent on emotional closeness for the stronger ties in the support group. Thus, for the purposes of our analyses, we simplified our categories of last contact into ‘weekly contact’ (merged categories 1–2) and ‘monthly contact (merged categories 1–4), as they overlap with the definitions of the support and sympathy group [40], and investigated the effect of emotional closeness on willingness to travel separately for these two categories.

In this study, we focus on face-to-face contact, as this has a genuine cost in travel time in a way that non-face-to-face contact (via e-mail, telephone etc.) does not. However, previous research on media multiplexity has demonstrated that the frequency of non-face-to-face communication is closely tied to the frequency of face-to-face communication [44] particularly for mobile phone use [45], [46]. Further, the level of emotional closeness is closely related to the frequency of both face-to-face and non face-to-face communication [16].

Distance was measured as how long (in minutes) it took the participant to travel to meet the contact, as reported by the participant (Minimum: 1; Maximum: 4800 mins; Table 1). Participants were instructed to list 0 mins. for individuals who were living with them, so cohabiting family were excluded. All responses greater than 1440 mins. (24 h) were recoded to a standard 1440 mins. We chose time, rather than a measure in kilometres, as our previous work suggests that individuals find it easier to estimate time rather than distance for network ties [15], [47].

**Table 1:** Descriptive statistics.

Variable	Relatedness	N	%
	0.03125	112	2.3
	0.125	1412	29.0
	0.25	2336	48.0
	0.5	1007	20.7

Variable	Contact	N	%
	in last 2 days	685	14.1
	between 3 and 7 days ago	569	11.7
	between 8 and 14 days ago	337	6.9
	between 15 and 30 days ago	450	9.2
	over a month ago	2780	57.1
	Never	46	.9

Variable	Distance	M	SD
	(mins.)	197	240

Variable	Emotional closeness	M	SD
	(score from 0 to 10)	5.67	2.67

Respondents also listed how emotionally close they felt to each biological relative on a scale from 1 to 10 (with 10 being very close). The (translated) question was formulated as follows: “*On a scale of 1–10* ***(where 10 is very close)*** *please say how close the person is to you in terms of how you feel about them.*” Very rarely participants listed 0, we have chosen to keep these data in the dataset (N = 48; 1% of total data). A single-item measure of emotional closeness has been used in a large number of previous studies by different research groups [13]–[15], [48]–[50] and is simple for participants to answer for a large number of network members. Further, a measure of emotional closeness has been shown to be the most reliable indicator of tie strength, as compared to other measures such as the duration of the relationship, the frequency of contact or the type of the relationship [51].

### Statistics

After reporting the descriptive statistics of our sample, we present the results of stratified Cox regression models, also known as stratified proportional hazard models [52]–[55] (see [56], [57] for examples of Cox regression models). This technique allows us to test whether, with increasing distance, more closely related individuals are more likely to maintain contact than more distantly related individuals. Cox regression is typically used to analyze the likelihood of survival over time [54], but has also been used to investigate the likelihood of an event, here, maintaining contact with increasing distance [58]. As detailed above, we built separate models for weekly and monthly contact (respectively: codes 1–2 and codes 1,2,3,4 coded as event). As is standard practice with Cox regression models, kin members contacted over a month ago or never were included in the models.

Cox regression makes relatively few assumptions compared to other statistical techniques, but a key one, which we tested, is the proportional hazard assumption. In our case, the key variables of interest (coefficient of relatedness, emotional closeness) should not be related to travel time [54]. Rephrased, it is possible that the relationship between willingness to travel and relatedness and/or emotional closeness is driven by a spurious relationship with distance. If this is the case then there will be evidence for significant ‘distance×relatedness’ and/or ‘distance×emotional closeness’ covariates and the model can be appropriately adjusted by including such a distance-dependent covariate.

Stratified Cox regression generates a hazard function for each participant and thus takes into account the nested structure of the data (multiple kinship ties are nested within each participant, and thus each kinship tie cannot be treated as an independent data point). We report coefficients from the Cox regression, the hazard ratios (*Exp(B)*) and the Wald statistics. For ease of interpretation, all coefficients were recoded in the same direction, so that a hazard ratio larger than 1 means a greater willingness to travel further to visit more closely related kin, as compared to more distantly related kin. A hazard ratio smaller than 1 means a greater willingness to travel further to visit more distantly related kin as compared to more closely related kin.

There were no control variables (such as nationality, age or gender) at participant level as these variables were either constant or a linearly dependent function of the stratum effect. For graphical representation, we present the aggregated data: the cumulative likelihood of travelling further by varying degrees of relatedness (rather than these functions for every single individual). As detailed above, we built separate models for weekly and monthly contact.

All analyses were conducted in SPSS 16.0 [59] and more details on the algorithms for (stratified) Cox Regression can be found in the SPSS manual [60] or in the cited works above.

## Results

Table 1 gives the descriptive statistics for all variables used in the analyses.

### Maintaining contact at least weekly

**Figure 1:**
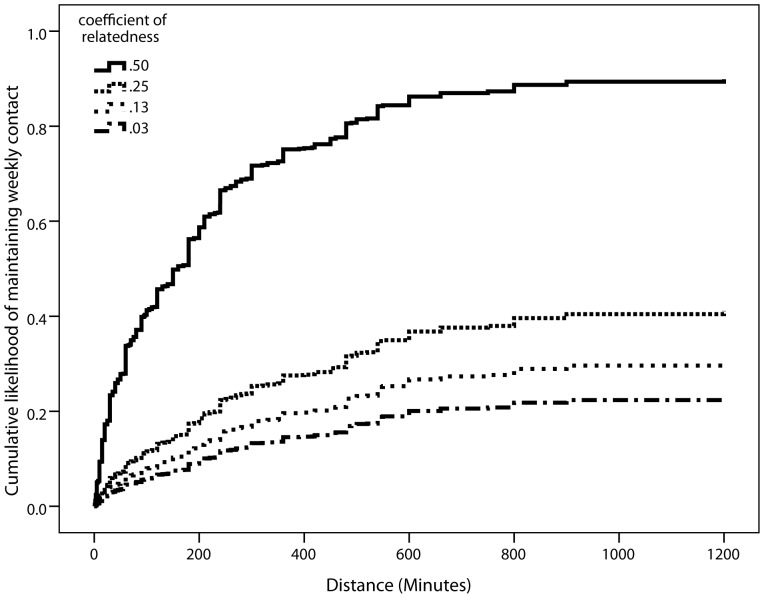
Cumulative likelihood of maintaining weekly contact (**Table 2**, Model 1), aggregated data.

Model 1 shows the coefficients for every comparison between categories of relatedness (Table 2). The Exp(B) in the tables are the hazard ratios. These can be converted into probabilities using the formula: probability = Exp(B)/(1+Exp(B)) [61]. For example, a hazard ratio of 11.36 (comparison of *r* = .5 to *r* = .032; Table 2), means that in 92% of the cases, individuals related at *r* = .5 travelled further to maintain weekly contact than those of who are related with *r* = .03. The coefficients for all the comparisons, with the exception of *r* = .03 vs. *r* = .125, were significant and sizeable. Figure 1 displays the pattern graphically: for weekly contact, individuals were more likely to travel further to visit more closely related kin, as compared to more distantly related kin.

**Table 2:** Coefficients and concomitant statistics for two stratified Cox Regressions for weekly contact as a function of distance.

MODEL 1 (−2LL = 5096)					
Relatedness (.5)	B	SE	Wald	Exp(B)	p
0.03125	2.430	0.373	42.510	11.358	<.00001
0.125	1.948	0.099	385.035	7.018	<.00001
0.25	1.531	0.077	393.269	4.621	<.00001

Relatedness (.25)	B	SE	Wald	Exp(B)	p
0.03125	0.899	0.372	5.848	2.458	.016
0.125	0.418	0.100	17.611	1.519	.00003

Relatedness (.125)	B	SE	Wald	Exp(B)	p
0.03125	0.481	0.374	1.654	1.618	.198

MODEL 2 (−2LL = 4878)					
Emotional closeness	B	SE	Wald	Exp(B)	p
(one unit)	0.312	0.023	188.220	1.366	<.00001

Relatedness (.5)	B	SE	Wald	Exp(B)	P
0.03125	0.931	0.391	5.670	2.538	.017
0.125	0.827	0.125	43.958	2.286	<.00001
0.25	0.680	0.095	51.211	1.973	<.00001

Relatedness (.25)	B	SE	Wald	Exp(B)	P
0.03125	0.252	0.380	5.848	1.286	.508
0.125	0.147	0.102	17.611	1.159	.150

Relatedness (.125)	B	SE	Wald	Exp(B)	P
0.03125	0.104	0.381	0.075	1.110	.784

(B = coefficient; SE = standard error; Wald = Wald test statistic; Exp(B) = Hazard ratio; p = p value associated with Wald test).

Model 1 could not be improved by adding a distance-dependent covariate (distance×coefficient relatedness: *p*>.1; see Text S1)

Model 2 includes the participant's rating of emotional closeness to the individual concerned (known as a ‘tie’ in network terminology). Overall, participants were willing to travel further to maintain contact with those who are emotionally closer to them (*Exp(B)* = 1.37; *p*<.0001). However, only the comparisons with *r* = .5 and other kin categories remained significant. Individuals were significantly more likely to travel to maintain weekly contact with those of *r* = .5, as compared to other categories of relatedness, even when emotional closeness is controlled for. Other comparisons between categories in Model 2 were no longer significant. This suggests that emotional closeness mediates the willingness to travel further to keep weekly contact for these kin categories. Given that the coefficients for *r* = .5 in comparison to other categories of kin remain significant, whereas for other comparisons between kin categories the coefficients became non-significant, it appears that parent-offspring and full sibling ties are substantially different from other biological kinship ties (such as grandparents or cousins), even after taking into account the level of emotional closeness.

Model 2 could be slightly improved by including a distance×emotional closeness interaction (*Wald* = 5.299; *p* = .021). A model with this interaction yielded a slightly stronger hazard ratio for emotional closeness than those reported in Table 2 (*Exp(B)* = 1.42). The significance and size of the other effects remained virtually unaltered.

### Maintaining contact at least once a month

**Figure 2:**
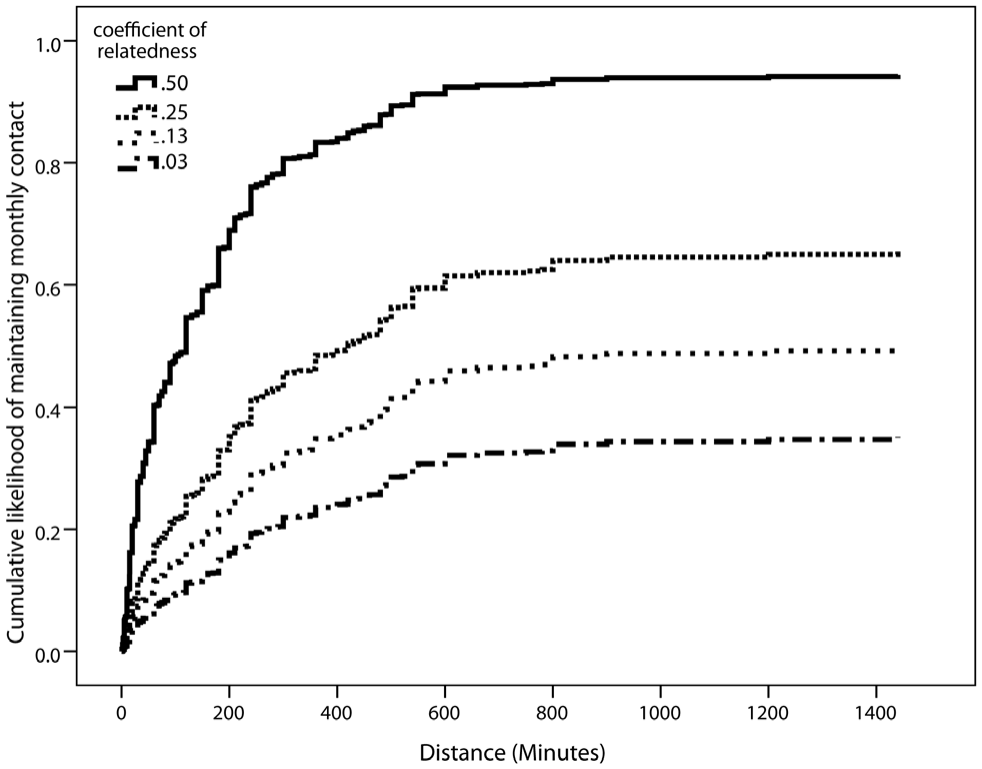
Cumulative likelihood of maintaining monthly contact (**Table 3**, Model 3), aggregated data.

Model 3 shows the coefficients for every comparison between categories of relatedness (Table 3; Figure 2). All coefficients, with the exception of *r* = .03 vs. *r* = .125, were significant and sizeable. The coefficient of *r* = .03 vs. *r* = .125 was, however, marginally significant (*p* = .06) and in the predicted direction. Figure 2 displays the pattern graphically for monthly contact: participants were more likely to travel further to visit more closely related kin, as compared to more distantly related kin. Model 3 could not be improved by adding a distance×coefficient of relatedness interaction (*p*>.1; see Text S1).

**Table 3:** Coefficients and concomitant statistics for two stratified Cox Regressions for monthly contact as a function of distance.

MODEL 3 (−2LL = 8728)					
Relatedness (.5)	B	SE	Wald	Exp(B)	P
0.03125	2.055	0.280	53.716	7.810	<.00001
0.125	1.532	0.077	396.378	4.627	<.00001
0.25	1.122	0.060	344.098	3.070	<.00001

Relatedness (.25)	B	SE	Wald	Exp(B)	P
0.03125	0.934	0.279	11.230	2.544	.001
0.125	0.410	0.073	31.578	1.507	<.00001

Relatedness (.125)	B	SE	Wald	Exp(B)	P
0.03125	0.524	0.280	3.508	1.688	.061

MODEL 4 (−2LL = 8471)					
Emotional closeness	B	SE	Wald	Exp(B)	P
(one unit)	0.252	0.017	226.930	1.287	<.00001

Relatedness (.5)	B	SE	Wald	Exp(B)	P
0.03125	0.836	0.295	8.037	2.308	.005
0.125	0.601	0.097	38.356	1.823	<.00001
0.25	0.405	0.075	29.168	1.500	<.00001

Relatedness (.25)	B	SE	Wald	Exp(B)	P
0.03125	0.431	0.285	2.289	1.539	.130
0.125	0.195	0.075	6.795	1.216	.009

Relatedness (.125)	B	SE	Wald	Exp(B)	P
0.03125	0.236	0.284	0.687	1.266	.407

(−2LL = −2LogLikelihood of the model; B = coefficient; SE = standard error; Wald = Wald test statistic; Exp(B) = Hazard ratio; p = p value associated with Wald test).

As with Model 2 for weekly contact, in Model 4 highly related kin (*r* = .5) were willing to travel further to maintain monthly contact than more distantly related kin, even after controlling for emotional closeness. The only other significant comparison was between *r* = .25 and *r* = .125. As with Model 2, in Model 4 the coefficients were greatly reduced in size, suggesting that emotional closeness mediates the effect of relatedness on the willingness to travel to keep in contact.

Model 4 could be slightly improved by adding a distance×emotional closeness interaction (*Wald* = 4.098; *p* = .043). This leads to a slightly stronger effect of emotional closeness (*Exp(B)* = 1.32). The comparisons for different categories of relatedness remain virtually unaltered.

## Discussion

Here, we provide the first test of the hypothesis that emotional closeness mediates the relationship between genetic relatedness and investment in kin using a real-life costly behaviour (travel time). In doing so, we integrate evolutionary theory and social psychology by exploring how inclusive fitness theory is mediated by psychological variables. There were three key results. First, exactly in line with predictions derived from inclusive fitness theory [1], individuals were willing to travel for longer to see more closely related kin (e.g. to see a parent or sibling, compared to an uncle or aunt). Second, when emotional closeness was included in the models, the comparisons between all categories of kin except for parents/children/siblings (*r* = .5) were strongly reduced in size and no longer significant, suggesting that the relationship between genetic relatedness and willingness to travel is mediated by emotional closeness for distantly related kin. Finally, and most importantly, even when controlling for emotional closeness, individuals were still willing to travel significantly further to see their closest relatives (*r* = .5) as compared to any other relatives.

In part, our results support the hypothesis that emotional closeness acts as a crucial mediating variable between genetic relatedness and altruism [13], [14]. Thus, willingness to travel further or for longer to see more distantly related kin appears to be driven by emotional closeness, rather than genetic relatedness: when emotional closeness was controlled for, the differences between distantly related kin were no longer significant. However, the results also add a crucial caveat to this hypothesis, namely that the mediating effect of emotional closeness appears to act differentially with respect to the level of genetic relatedness, with a clear distinction between the closest kin and more distant kin. Thus, our results suggest that rather than the psychological mechanisms underpinning kinship acting equally on all types of kin, in fact these psychological mechanisms may be distinct for different categories of kin and not be fully explained by emotional closeness. Emotional closeness appears to mediate investment for distantly related kin [13], [14], but there is a residual ‘kinship premium’ [12] for the most closely related kin. Future research could use the memory confusion paradigm [22] to examine whether people form separate implicit concepts of close and distant kin.

In this study, we used the variable emotional closeness to measure the strength of the social bond between two individuals. This measure has been widely used in social psychology [14]–[18], [26], is the most reliable indicator of tie strength [51] and correlates highly with self-reported altruism [12]. However, despite its wide use, there is no definitive definition of emotional closeness (see e.g., [20] p. 17), and future work could usefully unpack the one-dimensional concept of emotional closeness in more detail. Further, the single-item measure of emotional closeness used in this and many other studies [13]–[15], [50], [62], [63] may be a somewhat imprecise measure of the emotional intensity of the relationship, as compared to more detailed assessments of the relationship, for example based on interviews (e.g., [64]). The fact that this somewhat imprecise and ‘noisy’ measure mediated most of the relationship between the degree of relatedness and willingness to travel lends support to the theory that emotional closeness is an important mediator of altruism to kin, with the crucial exception of the most closely related kin (*r* = .5).

In relation to cooperation and altruism, it may be that emotional closeness tracks the degree to which past altruistic behaviour has been reciprocated, i.e. whether the cost of the altruistic behaviour has been balanced by the benefits received [20]. Thus, emotional closeness may be a component of ‘attitudinal reciprocity’ [65] or ‘emotional bookkeeping’ [66] as used in the animal literature, particularly given the working memory constraints of keeping track of past interactions [67], [68]. Moreover there might be other cues to kinship which could be specific to certain kinship ties. Lieberman and colleagues have argued for example that both co-residence duration and the maternal perinatal association are particularly important for kin recognition among siblings [23]. Other research has pointed to the role of facial resemblance for kin recognition (e.g., [21], [69], [70] review in [71]) and the role of psychological similarity (e.g., [72]). These other cues to kinship, such as physical and psychological similarity, could be driving the difference between close kin (*r* = .5) as opposed to other kin categories and which cue is predominant could be contingent on many factors, such as sex (e.g., [73]) and type of kin (e.g., [23], [74]). Moreover these kinship cues could interact as suggested by work on sexual imprinting: a preference for a self-resembling partner can be a function of the relationship with one's parents (e.g., [75], [76], but see [77]).

Whilst this study benefitted from the use of a large non-student sample [78], it did have some limitations. First, it is possible that the difference between parent-child and sibling relationships versus other kinship relationships found in our study are due to the uni-dimensional nature of our measure of emotional closeness. On the other hand, as outlined above, it is actually all the more surprising that a uni-dimensional measure mediates most of the relationship between relatedness and willingness to travel. Nonetheless, further research is necessary into the proximate factors beyond emotional closeness affecting the likelihood of visiting kin of different levels of genetic relatedness. For example, perceived obligation varies with genetic relatedness and has been shown to influence helping behaviour towards kin [14]. Second, participants were asked to self-report travel time and journeys of the same travel time may have different monetary costs, depending on the mode of transport used. Third, there are other factors, apart from biological relatedness, that influence the willingness to travel to meet up with certain kin, which we did not measure such as, for example, how rewarding the visit is, or whether the kin are matrilineal or patrilineal (e.g., [58]). Fourth, the current research, did not investigate who initiates contact which is asymmetric for many kinship categories (e.g. parent-offspring, grandparent-grandchild). For many kinship categories, there are differences in reproductive value (the expected future contribution to one's fitness [79]), and reproductive value has been shown to predict asymmetries in kin investment among humans [47]. For example, childless individuals report feeling closer to nieces/nephews than to aunts/uncles [47]. In certain cases, reproductive value will be an even more important factor for kin investment than biological relatedness (e.g. [80]). Nonetheless, *all else being equal*, from an ultimate perspective we expect individuals to discriminate between kin relationships which differ according to the coefficient of relatedness and our data suggest this is indeed the case in this sample. Fifth, it is possible that there are some limitations inherent to our statistical analyses. One issue could be that our model assumes statistical independence in contact and that in reality when individuals have contact with several kin at the same time (for example for Easter, a baptism or a birthday). This is indeed possible, but if it was driving the effect, then it should have made it harder to find the effects for relatedness in Models 1 and 3. In addition, the number of ‘events’ (weekly or monthly contact) per individual might have been relatively low, which could potentially lead to biases in the stratified Cox regression test statistics. However, a low frequency likely would increase Type 1 errors making it harder to find the results detailed above, and simulations suggest that Cox regression performs relatively well even when the number of events are low [81].

Finally, our correlational design cannot indicate the directionality of the relationship between emotional closeness and investment in kin: does contact with kin lead to emotional attachment, or does emotional attachment lead to contact? Previous longitudinal research has suggested that contact frequency and emotional closeness are very closely temporally linked [16], so fine grained data using electronic communication records (e.g., mobile phone records [45]) may be more effective at disentangling these two possibilities than even longitudinal questionnaire studies. Further studies are clearly needed in order to fully understand the dynamics of investment in kin relationships.

## Conclusion

In this paper, we examined a real-life costly behaviour and demonstrated that for distantly related kin, emotional closeness mediated the relationship between genetic relatedness and willingness to travel. However, even when controlling for emotional closeness, individuals were still willing to travel significantly further to see their closest relatives (parents, children and siblings), as compared to any other relatives. Thus, the way in which emotional closeness mediates investment appears to operate differently across different kinship categories, in that it has a stronger mediating effect on distantly related kin, as compared to more closely related kin.

## Supporting Information

Text S1 **Additional information on models with time dependent covariates.**
(DOCX)
