# Supplementary material for: Going That Extra Mile: Individuals Travel Further to Maintain Face-to-Face Contact with Highly Related Kin than with Less Related Kin
Source: PLoS One. 2013 Jan 25;8(1):e53929. doi: 10.1371/journal.pone.0053929 (PMC3556071; doi:10.1371/journal.pone.0053929)
Supplement: Text S1 — Additional information on models with time dependent covariates. (DOCX) [file pone.0053929.s001.docx]

Additional information on models with time dependent covariates

The proportional hazards assumption was tested with SPSS 16.0 Cox regression with time-dependent covariates, we specified the time-dependent covariate is specified T_COV as Time*(coeffrelat) and both coeffrelat (as a categorical) as predictors. These are the results reported in the text.

We also used a dummy coded approach for the time-dependent covariates (relatedness.5vsother; relatedness.25vsother; relatedness.125vsother). When sequentially testing all interactions between time and a dummy, none of the models could be improved (all p>.09). This suggests that the results are not driven by an interaction between time and a particular kin category.

We also tested an approach where the time-dependent covariate was specified as (Time* relatedness.5vsother + Time* relatedness.25vsother + Time* relatedness.125vsother). These models did show evidence a time-dependent covariate as significant at p=.007 and p=.009, for Model 1 and Model 3 respectively. However, inclusion of these time dependent covariates, leads to similar, if not stronger, results as those reported in the text (all Model 1: all p<.017; Model 3: all p<.006). Given that it is unclear if the time dependent covariate should be modeled as a simple linear function of the dummies (1) and it is hard to determine the meaning of the hazard ratio when this covariate is included (2), we have opted to report the model without this time-dependent covariate. Nonetheless, these results suggest that accounting for time-dependence of kin categories leads to similar, if not stronger results.
